# Supplementary material for: Floquet-enhanced spin swaps
Source: Nat Commun. 2021 Apr 9;12:2142. doi: 10.1038/s41467-021-22415-6 (PMC8035411; doi:10.1038/s41467-021-22415-6)
Supplement: Supplementary file 1 — Supplementary Information [file 41467_2021_22415_MOESM1_ESM.pdf]

Supplementary Information for Floquet-Enhanced Spin Swaps

Haifeng Qiao,[1] Yadav P. Kandel,[1] John S. Van Dyke,[2] Saeed Fallahi,[3,4] Geoffrey C. Gardner,[4,5] Michael J. Manfra,[3,4,5,6] Edwin Barnes,[2] and John M. Nichol[1,*]

[1]*Department of Physics and Astronomy, University of Rochester, Rochester, NY, 14627 USA*

[2]*Department of Physics, Virginia Tech, Blacksburg, Virginia, 24061, USA*

[3]*Department of Physics and Astronomy, Purdue University, West Lafayette, IN, 47907 USA*

[4]*Birck Nanotechnology Center, Purdue University, West Lafayette, IN, 47907 USA*

[5]*School of Materials Engineering, Purdue University, West Lafayette, IN, 47907 USA*

[6]*School of Electrical and Computer Engineering, Purdue University, West Lafayette, IN, 47907 USA*

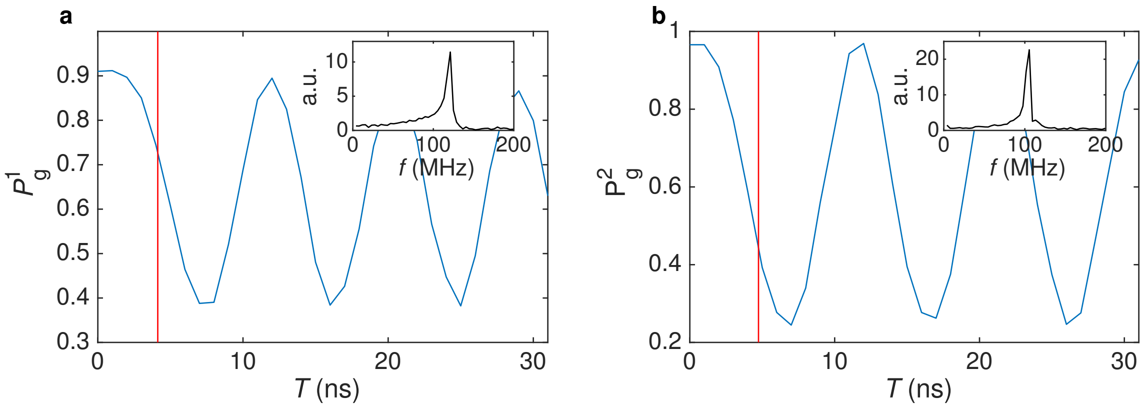

Supplementary Figure 1. (a) Exchange oscillations vs. evolution time (T) measured on the left pair. (b) Exchange oscillations vs. T measured on the right pair. In both panels, the red line indicates the expected π-pulse time based on the frequency of exchange oscillations. The frequency is chosen as the frequency giving the peak in the absolute value of the FFT of the data (insets). In both cases, the actual π-pulse time is longer than the expected value, because of the ns-level rise time in the pulse, leading to the observed frequency chirp.

* john.nichol@rochester.edu

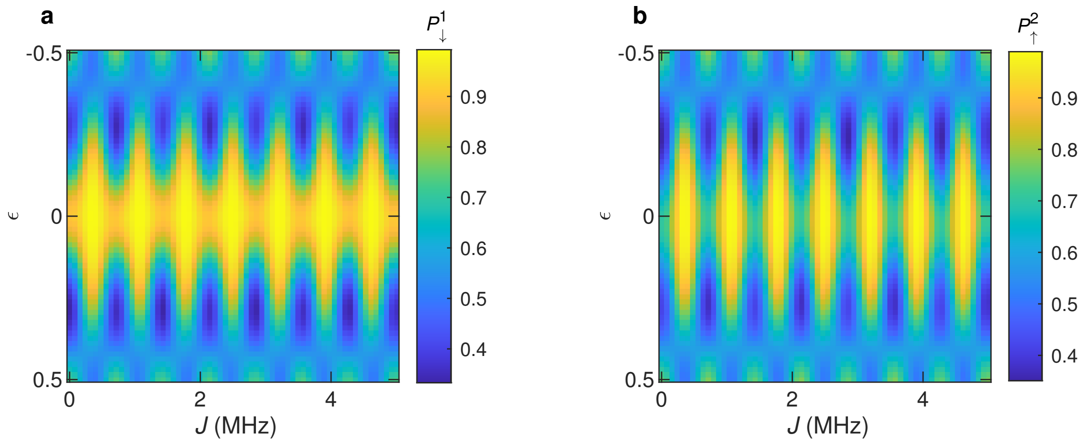

Supplementary Figure 2. Numerical simulation of an $N = 2$ Ising spin chain after 4 Floquet steps. The initial state is $|\downarrow\uparrow\rangle$. (a) Spin-down probability of the left spin. (b) Spin-up probability of the right spin. The simulations are averaged over 128 charge and hyperfine realizations.

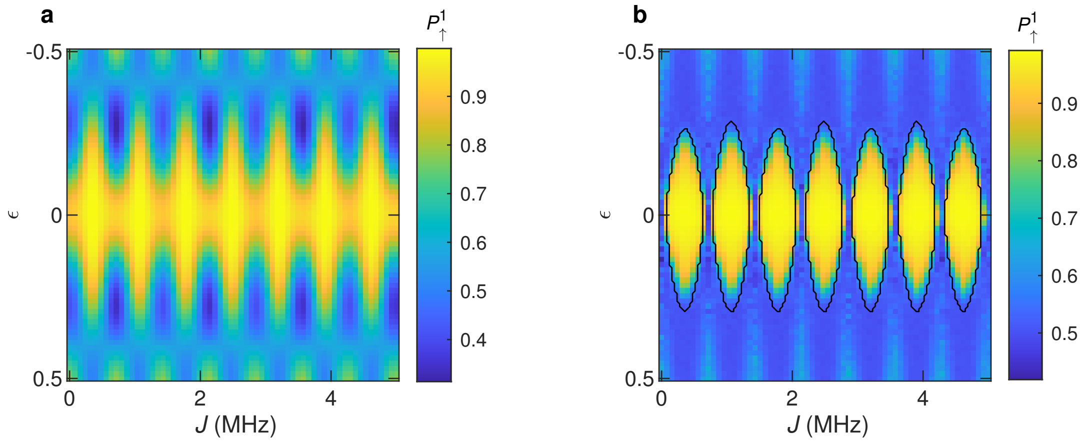

Supplementary Figure 3. Numerical simulation of an $N = 8$ Ising spin chain after (a) 4 and (b) 1024 Floquet steps. Both panels plot the probability to find the first spin in the $|\uparrow\rangle$ state. The initial state is $|\uparrow\downarrow\uparrow\downarrow\uparrow\downarrow\uparrow\downarrow\rangle$. In panel (b), the theoretical semiclassical DTC phase diagram is overlaid. The simulations are averaged over 128 charge and hyperfine realizations.
